# *PNPLA 3* I148M genetic variant associates with insulin resistance and baseline viral load in HCV genotype 2 but not in genotype 3 infection

**DOI:** 10.1186/1471-2350-13-82

**Published:** 2012-09-14

**Authors:** Karolina Rembeck, Cristina Maglio, Martin Lagging, Peer Brehm Christensen, Martti Färkkilä, Nina Langeland, Mads Rauning Buhl, Court Pedersen, Kristine Mørch, Gunnar Norkrans, Kristoffer Hellstrand, Magnus Lindh, Carlo Pirazzi, Maria Antonella Burza, Stefano Romeo, Johan Westin

**Affiliations:** 1 Department of Infectious Diseases/Virology, Institute of Biomedicine, University of Gothenburg, Gothenburg, Sweden; 2 Department of Molecular and Clinical Medicine and Center for Cardiovascular and Metabolic Research, the Sahlgrenska Academy, University of Gothenburg, Gothenburg, Sweden; 3 Department of Infectious Diseases, University of Southern Denmark, Odense, Denmark; 4 Department of Gastroenterology, Helsinki University, Helsinki, Finland; 5 Department of Medicine, Haukeland University Hospital and Institute of Medicine, University of Bergen, Bergen, Norway; 6 Department of Infectious Diseases, Aarhus University, Aarhus, Denmark

**Keywords:** Hepatitis C, *PNPLA 3*, Insulin resistance, Viral load

## Abstract

**Background:**

Hepatic steatosis in HCV patients has been postulated as a risk factor associated with a higher frequency of fibrosis and cirrhosis. A single genetic variant, *PNPLA3* I148M, has been widely associated with increased hepatic steatosis. Previous studies of the *PNPLA3* I148M sequence variant in HCV infected individuals have reported an association between this variant and prevalence of steatosis, fibrosis, and cirrhosis. To evaluate the impact of *PNPLA3* I148M variant on metabolic traits and treatment response in HCV genotype 2 and 3 infected patients.

**Methods:**

Three hundred and eighty-two treatment naïve HCV genotype 2 or 3 infected patients were included in a phase III, open label, randomized, multicenter, investigator-initiated trial (the NORDynamIC study), in which pretreatment liver biopsies were mandatory. *PNPLA3I148M* genotyping was performed in a total of 359 Caucasian patients.

**Results:**

In HCV genotype 2 infected patients carrying the *PNPLA3* 148M allele, there was significantly increased insulin resistance (P = 0.023) and lower viral load (P = 0.005) at baseline as well as the first seven days of antiviral treatment. These results were not observed in HCV genotype 3 infected patients.

**Conclusions:**

Our results suggest a possible association between the *PNPLA3* 148M allele and insulin resistance as well as baseline viral load in HCV genotype 2, but not in genotype 3.

## Background

Chronic hepatitis C virus (HCV) infection is a major cause of cirrhosis and liver failure [1]. Hepatic steatosis is twice as common in HCV infected patients as in the normal population [2] and it is associated with fibrosis and cirrhosis development [3,4]. Metabolic host factors, mainly obesity, insulin resistance and type 2 diabetes, are thought to play an important role in the steatosis development in non–genotype 3 infected individuals, whereas HCV genotype 3-associated hepatic steatosis appears to mainly result from a direct viral action [4-6].

A single genetic variant, I148M, entailing a change from isoleucine (I) to methionine (M) at position 148 in the human patatin-like phospholipase domain containing 3 gene (*PNPLA3*, Adiponutrin) on chromosome 22 has been widely associated with increased hepatic steatosis [7-11]. Recently, in a murine model, *PNPLA3* was found to be involved in the hepatic metabolism of triglycerides and in the regulation of systemic glucose homeostasis [12]. Even if previous reports failed to find an association with insulin resistance assessed by euglycemic hyperinsulinemic clamp or surrogates in humans [8,13-15], a recent study reported that *PNPLA3* I148M variant is associated with insulin resistance in a normoglycaemic population from Taiwan [16]. Furthermore, the *PNPLA3* 148M allele genetic variant was found to be associated with lower serum triglyceride and higher fasting glucose levels in individuals with gallstones [17]. In the setting of HCV infection, *PNPLA3* I148M sequence variant has been extensively associated with steatosis, fibrosis progression, cirrhosis and hepatocellular carcinoma [18-21]. So far, no association has been reported between the *PNPLA3* I148M variant and insulin resistance or triglyceride levels in individuals with HCV infection [18,20].

The aim of the present study was to examine the impact of *PNPLA3* I148M variant on metabolic traits and treatment response in a well phenotyped HCV genotype 2 and 3 cohort, within the framework of the NORDynamIC treatment trial [22].

## Methods

### Patients

Three hundred and eighty-two treatment naïve HCV genotype 2 or 3 infected patients were included in a phase III, open label, randomized, multicenter, investigator-initiated trial (the NORDynamIC study) conducted at 31 centers in Denmark, Finland, Norway, and Sweden. Details regarding demographics and clinical characteristics have been previously reported [22]. Briefly, all patients were adults with compensated liver disease, had detectable HCV RNA, and were seronegative for hepatitis B surface antigen and for antibodies to human immune deficiency virus. A liver biopsy consistent with chronic hepatitis C within 24 months prior to inclusion was also required. At study entry, patients were randomized to either 12 or 24 weeks of treatment with 180 μg of peg-interferon α-2a once weekly and 800 mg/day ribavirin. After excluding all non-Caucasians as well as two patients positive for both HCV genotypes 2 and 3, a total of 359 patients were examined. Alcohol consumption was measured as standard drink units (12 g of pure alcohol) per day [23].

### PCR *PNPLA3* genotyping, rs738409

The variation at *rs738409* was determined by amplification on an ABI7300 (Applied Biosystems) using *rs738409_F*, GTGCCTGTCGTGTACTGAACCA as forward primer, *rs738409_R*, AGCGCGGAGTGCAATTCA as reverse primer, and Taqman MGB (minor groove binding) probes (*rs738409-pC*, FAM-CTGCTTCATCCCCTTC-MGB, *rs738409-pG*, VIC-GGAAGGAGGGATAAGGCCACT-MGB) for allelic discrimination.

#### Serum metabolic parameters

Baseline fasting glucose (mmol/L) was measured at individual sites on freshly drawn serum, whereas fasting serum insulin was analyzed on frozen samples at the central laboratory (mU/L, Architect Insulin, Abbott, Abbott Park, IL). Diabetes mellitus was defined as fasting serum glucose ≥ 7.0 mmol/L. Insulin resistance was assessed by using the homeostatic model assessment insulin for resistance (HOMA-IR) index. The gold standard for insulin resistance assessment is the euglycemic hyperinsulinemic clamp technique [24]. However, this method is demanding and time-consuming and therefore is not commonly used in genetic and epidemiologic studies. The HOMA-IR is a well known and widely used surrogate index to quantify insulin resistance that is highly correlated with clamp estimations [25,26]. HOMA-IR was calculated using the formula: (Glucose mmol/L × Insulin mU/L)/22.5 [27].

### Histological assessment

Fibrosis and necroinflammatory activity were assessed according to the Ishak protocol by two experienced observers in a dual observer consensus fashion as described [22,28]. Specifically, presence of fibrosis was defined as Ishak stage ≥1, and cirrhosis as Ishak stage 5–6. Steatosis was graded as follows: absent (grade 0), mild (less than 30 % of hepatocytes involved, grade 1), moderate (30-70 % of hepatocytes involved, grade 2) or severe (>70 % of hepatocytes involved, grade 3), although in the present study reported as absent or present (grade 1–3).

### HCV RNA quantification

HCV RNA was determined by RT-PCR of plasma using Cobas AmpliPrep/COBAS TaqMan HCV Test (Roche Diagnostics, Branchburg, NJ), which quantifies HCV RNA with a limit of detection of 15 IU/mL. HCV RNA quantification was performed on days 0, 3, 7, 8, 29, and on weeks 8, 12, and 24 (for those receiving 24 weeks of therapy), and 24 weeks after completion of therapy. All samples were frozen (−70 °C), and subsequently analyzed at a central laboratory.

### Statistical methods

Continuous variables were presented as median and the range between the 25th and 75th percentile (interquartile range). Categorical variable distributions were compared using either the *χ*^2^ test or the Fisher’s exact test. Continuous variables were analyzed with linear regression, when necessary after logarithmic transformation in order to create a normal distribution. Metabolic parameters were adjusted for age, gender and body mass index (BMI). Both additive (*add*) and recessive (*rec*) inheritance models were tested. Binary logistic regression analysis was used to calculate allelic odds ratio and 95 % confidence interval. Frequency distribution of genetic variants was evaluated according to the Hardy Weinberg equilibrium. All statistical analyses were performed using the IBM SPSS statistics version 19 (IBM Corporation, Somers, NY) software package. A two-sided P-value of <0.05 was considered statistically significant.

### Ethical considerations

The Regional Ethics Review Board in Gothenburg approved the study. All patients signed informed consent. The study is registered at the NIH trial registry (ClinicalTrials.gov Identifier: NCT00143000).

## Results

### *PNPLA3* I148M genotype

A total of 359 individuals were analyzed for the *PNPLA3* I148M genotype (II, IM or MM). Among these, 103 were infected with HCV genotype 2 and 256 with genotype 3. The distribution of the *PNPLA3* I148M genotype was in Hardy Weinberg equilibrium (P = 0.691 for genotype 2 and P = 0.468 for genotype 3, Additional file 1: Table S1). The *PNPLA3* I148M genotype frequency in our study cohort ( Additional file 1: Table S1) was consistent with previously reported frequencies for Northern Europeans [29]. Study group characteristics stratified by HCV genotype 2 or HCV genotype 3 infection are described in Table 1. Individuals with HCV genotype 2 infection were on average nine years older, they had lower alanine transferase (ALT) levels and lower degree of steatosis compared to individuals with HCV genotype 3 infection. They also had higher total cholesterol and HCV RNA at baseline compared to the HCV genotype 3 group (Table 1). 

**Table 1:** Baseline characteristics stratified by HCV genotype

	**HCV genotype 2 n = 103**	**HCV genotype 3 n = 256**	**P value**
**Male gender, n (%)**	62 (60)	154 (60)	0.920
**Age, years**	49 (41–54)	40 (32–48)	<0.001
**BMI, kg/m**^**2**^	25^a^ (23–27)	25^b^ (23–28)	0.290
**ALT, U/l**	74^a^ (44–144)	108^a^ (66–175)	<0.001
**Total cholesterol, mmol/L**	4.6^c^ (4.1-5.4)	3.9^d^ (3.3-4.6)	<0.001
**Triglycerides, mmol/L**	0.9^c^ (0.7-1.3)	0.9^d^ (0.7-1.4)	0.748
**Glucose, mmol/L**	4.9^d^ (4.6-5.4)	4.9^e^ (4.5-5.4)	0.507
**Insulin, mIU/L**	12^f^ (6–24)	12^e^ (7–26)	0.458
**HOMA-IR, U**	2.5^g^ (1.3-6.0)	2.7^h^ (1.6-5.9)	0.088
**Baseline HCV RNA, log**_**10**_**IU/mL**	6.5 (5.8-6.8)	6.1 (5.4-6.7)	0.025
**Steatosis**^**1**^**, n (%)**	49^c^ (50)	171^i^ (73)	<0.001
**Cirrhosis**^**2**^**, n (%)**	14^c^ (14)	29^i^ (12)	0.807
**Diabetes mellitus, n (%)**	4^d^ (4)	7^e^ (3)	0.823

Categorical traits, expressed as number (n) and relative proportion (%), have been compared by *χ*^2^ test. Continuous traits are expressed as median (interquartile range) and have been analyzed using a linear regression model. For additional information on statistical analyses, see methods.

^1^Steatosis was defined as histological grade > 0. ^2^Cirrhosis was defined as Ishak stage 5–6.

Missing data: ^a^n = 1, ^b^n = 8, ^c^n = 4, ^d^n = 6, ^e^n = 15, ^f^n = 7, ^g^n = 13, ^h^n = 27,^i^n = 23.

*Abbreviations:* HCV, hepatitis C virus; BMI, body mass index; ALT, alanine transferase; HOMA-IR, homeostasis model assessment for insulin resistance; II, individuals with two 148I alleles; MM, individuals with two 148 M alleles; IM, heterozygotes.

### Clinical and biochemical profile

**Figure 1:**
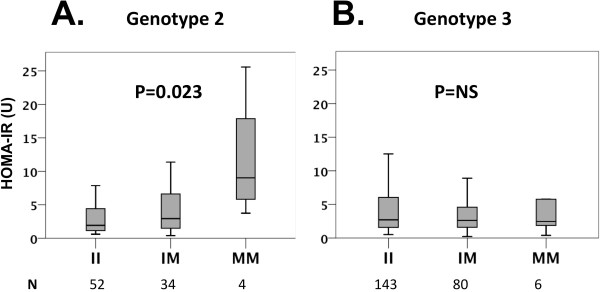
**Homeostasis model assessment for insulin resistance (HOMA-IR) in HCV genotype 2 (A) and genotype 3 (B) infected patients, stratified by the *PNPLA3 *I148M genotype.** P values were calculated using linear regression after logarithmic transformation of HOMA-IR values and adjusted for age, gender and BMI.

*PNPLA3* 148M allele carriers were found to have increased insulin resistance in HCV genotype 2 infected patients (P*add* = 0.023, P*rec* = 0.005; Table 2 and Figure 1 A). No association with insulin resistance and the *PNPLA3* 148M allele was found in individuals with HCV genotype 3 (Table 3 and Figure 1 B). Results were virtually identical after excluding individuals with diabetes mellitus (HCV genotype 2 HOMA-IR (U): II 1.8 [1.1-4.2], IM 3.0 [1.4-6.7], MM 9.0 [4.8-21.7]; P*add* = 0.003, P*rec* = 0.002; HCV genotype 3 HOMA-IR (U): II 2.6 [1.5-5.8], IM 2.6 [1.6-4.8], MM 2.5 [1.5-8.4]; P*add* = 0.811, P*rec* = 0.973). No significant association of triglyceride levels with *PNPLA3* I148M genotypes was found in either HCV genotype 2 or 3 patients (Tables 2 and 3). In HCV genotype 2 individuals, the *PNPLA3* 148M allele was found to be associated with higher ALT levels only if analyzed under a recessive inheritance model (P*rec* = 0.045, Table 2). Carriage of the *PNPLA3* 148M allele was not associated with age, gender, BMI or total cholesterol in either HCV genotype group (Tables 2 and 3).

**Table 2:** **Baseline characteristics according to** ***PNPLA3*** **I148M sequence variant in HCV genotype 2 patients**

	**II**	**IM**	**MM**	**P value**	**P value**
	**n = 56**	**n = 43**	**n = 4**	***additive***	***recessive***
**Male gender, n (%)**	33 (59)	27 (63)	2 (50)	0.830	0.999
**Age, years**	50 (42–56)	48 (37–54)	51 (36–53)	0.370	0.887
**BMI, kg/m**^**2**^	25 (23–27)	25^a^ (22–27)	23 (21–33)	0.773	0.981
**ALT, U/l**	73^a^ (44–150)	72 (39–137)	147 (88–455)	0.406	0.045
**Total cholesterol, mmol/L**	4.8^b^ (4.1-5.5)	4.6^a^ (4.2-5.1)	4.2 (3.9-5.8)	0.283	0.756
**Triglycerides, mmol/L**	1.0^b^ (0.8-1.3)	0.9^a^ (0.7-1.3)	0.7 (0.4-1.3)	0.094	0.065
**Glucose, mmol/L**	4.9^d^ (4.6-5.1)	5.0^e^ (4.5-5.4)	4.5 (4.2-5.6)	0.502	0.724
**Insulin, mIU/L**	10^d^ (5–23)	13^c^ (7–29)	48 (24–86)	0.027	0.001
**HOMA-IR, U**	1.9^e^ (1.1-4.5)	2.9^f^ (1.4-6.7)	9.0 (4.8-21.7)	0.023	0.005
**Baseline HCV RNA, log**_**10**_**IU/mL**	6.5 (6.0-6.9)	6.4 (5.3-6.8)	5.3 (4.7-6.4)	0.005	0.073
**Steatosis**^**1**^**, n (%)**	23^b^ (43)	23^a^ (55)	3 (75)	0.327	0.362
**Cirrhosis**^**2**^**, n (%)**	7^b^ (13)	6^a^ (14)	1 (25)	0.669	0.462
**Diabetes mellitus, n (%)**	3^d^ (6)	1^e^ (3)	0 (0)	0.694	0.998

Categorical traits, expressed as number (n) and relative proportion (%), have been compared by Fisher exact test. Continuous traits are expressed as median (interquartile range) and have been analyzed using a linear regression model. For additional information on statistical analyses, see methods.

^1^Steatosis was defined as histological grade > 0. ^2^Cirrhosis was defined as Ishak stage 5–6.

Missing data: ^a^n = 1, ^b^n = 3, ^c^n = 5, ^d^n = 2, ^e^n = 4, ^f^n = 9.

*Abbreviations:* PNPLA3, patatin-like phospholipase domain-containing 3; HCV, hepatitis C virus; BMI, body mass index; ALT, alanine transferase; HOMA-IR, homeostasis model assessment for insulin resistance; II, individuals with two 148I alleles; MM, individuals with two 148 M alleles; IM, heterozygotes.

**Table 3:** **Baseline characteristics according to** ***PNPLA3*** **I148M sequence variant in HCV genotype 3 patients**

	**II**	**IM**	**MM**	**P value**	**P value**
	**n = 159**	**n = 91**	**n = 6**	***additive***	***recessive***
**Male gender, n (%)**	97 (61)	52 (57)	5 (83)	0.466	0.407
**Age, years**	39 (31–47)	41 (35–48)	42 (29–46)	0.128	0.653
**BMI, kg/m**^**2**^	26^a^ (23–29)	25^a^ (23–28)	26 (22–28)	0.232	0.644
**ALT, U/l**	108 (68–175)	106^d^ (62–181)	69 (61–109)	0.398	0.248
**Total cholesterol, mmol/L**	4.0^e^ (3.3-4.7)	3.8^a^ (3.2-4.5)	3.7 (3.1-5.7)	0.909	0.452
**Triglycerides, mmol/L**	0.9^e^ (0.7-1.3)	0.9^a^ (0.7-1.4)	0.8 (0.6-1.1)	0.336	0.256
**Glucose, mmol/L**	4.9^c^ (4.5-5.6)	4.9^f^ (4.6-5.3)	4.8 (4.6-5.4)	0.147	0.882
**Insulin, mIU/L**	13^g^ (7–26)	12^h^ (7–25)	12 (6–39)	0.800	0.785
**HOMA-IR, U**	2.7^i^ (1.5-6.1)	2.6^j^ (1.6-4.7)	2.5 (1.5-8.4)	0.882	0.941
**Baseline HCV RNA, log**_**10**_**IU/mL**	6.0 (5.3-6.7)	6.2 (5.6-6.7)	6.8 (6.1-7.1)	0.030	0.100
**Steatosis**^**1**^**, n (%)**	99^k^ (68)	68^c^ (82)	4^d^ (80)	0.068	0.989
**Cirrhosis**^**3**^**, n (%)**	17^k^ (12)	11^c^ (13)	1^d^ (20)	0.636	0.489
**Diabetes mellitus, n (%)**	6^c^ (4)	1^f^ (1)	0 (0)	0.520	0.996

Categorical traits, expressed as number (n) and relative proportion (%), have been compared by Fisher exact test. Continuous traits are expressed as median (interquartile range) and have been analyzed using a linear regression model. For additional information on statistical analyses, see methods.

^1^Steatosis was defined as histological grade > 0. ^2^Cirrhosis was defined as Ishak stage 5–6.

Missing data: ^a^n = 4, ^b^n = 18, ^c^n = 8, ^d^n = 1, ^e^n = 2, ^f^n = 7, ^g^n = 9, ^h^n = 6, ^i^n = 16, ^j^n = 11, ^k^n = 14. *Abbreviations:* PNPLA3, patatin-like phospholipase domain-containing 3; HCV, hepatitis C virus; BMI, body mass index; ALT, alanine transferase; HOMA-IR, homeostasis model assessment for insulin resistance; II, individuals with two 148I alleles; MM, individuals with two 148 M alleles; IM, heterozygotes.

### Viral kinetics

**Figure 2:**
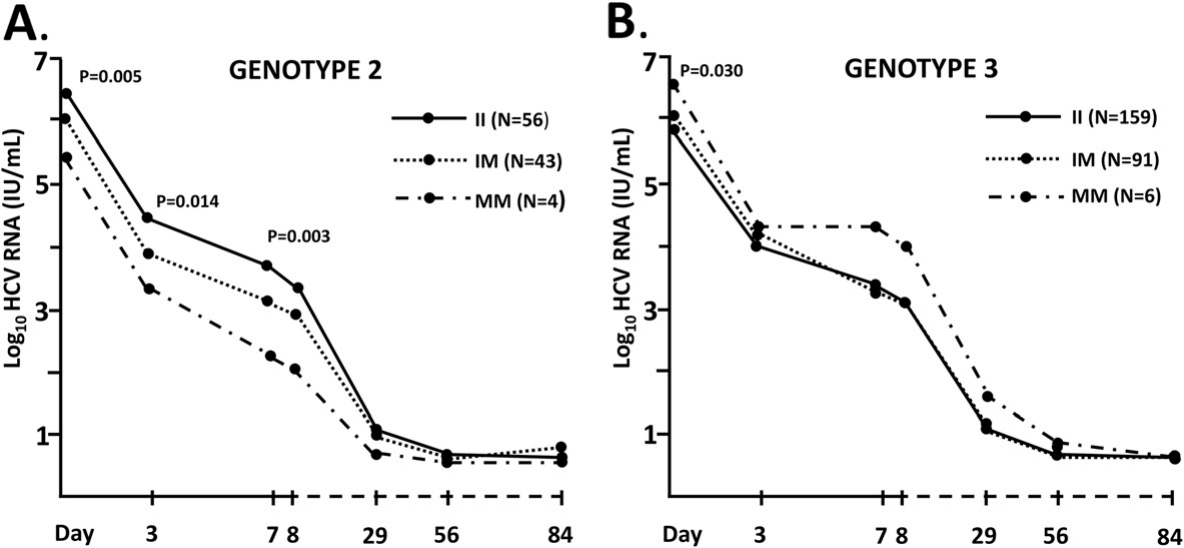
**HCV viral load according to the *PNPLA3 *I148M genotype in HCV genotype 2 (A) and genotype 3 (B) infected patients from start of treatment (day 0) through treatment week 12 (day 84).** Viral load is expressed as mean log_10_ value at each time point. P values were calculated for the difference in viral load across *PNPLA3* I148M genotypes at each time point using linear regression.

Among the HCV genotype 2 infected individuals, the *PNPLA3* 148M allele was associated with lower viral load at baseline (P*add* = 0.005, P*rec* = 0.073, Table 2), on day 3 (P*add* = 0.014 P*rec* = 0.130) and on day 7 (P*add* = 0.003, P*rec* = 0.036; Figure 2A). In contrast, HCV genotype 3 patients with the *PNPLA3* 148M allele genotype had higher baseline viral load (P*add* = 0.030, P*rec* = 0.100, Table 3). No further associations between the *PNPLA3* genotype and viral load during treatment (Figure 2B), first or second phase decline in HCV RNA, or final treatment outcome (sustained viral response rate in the HCV genotype 2 group was 66, 70 and 100 % and in the HCV genotype 3 group 67, 74 and 67 %, respectively in the II, IM and MM groups respectively), were observed.

### Liver histology

A trend, although non-significant, towards a higher prevalence of steatosis was observed in *PNPLA3* 148M allele carriers in both HCV genotype 2 and 3 subjects (Tables 2 and 3). *PNPLA3* 148M allele was found associated with hepatic steatosis increased risk, although non-significant, in HCV genotype 2 (allelic Odds Ratio, O.R. = 2.3, 95 % Confidence Interval C.I. = 0.9-5.5, P*add* = 0.053, P*rec* = 0.154, after adjustment for age, gender and BMI) and 3 (allelic O.R. = 1.7, 95 % C.I. = 0.9-3.4, P*add* = 0.096, P*rec* = 0.991, after adjustment for age, gender and BMI) subjects. No association between the prevalence of cirrhosis and the *PNPLA3* 148M allele was observed in either HCV genotype 2 or 3 infected subjects (Tables 2 and 3).

## Discussion

The main finding of the present study is the association between the *PNPLA3* 148M allele and increased insulin resistance and lower baseline viral load among HCV genotype 2 infected patients. *PNPLA3* I148M is the most widely replicated genetic variant associated with increased hepatic steatosis [7-11,30]. Moreover, *PNPLA3* 148M allele carriers with HCV infection have been reported to have increased prevalence of steatosis, fibrosis, cirrhosis and hepatocellular carcinoma [18-21]. Recent studies on the *PNPLA3* 148M allele and glucose metabolism suggest a possible involvement of this allele as a genetic determinant [16,17].

In this study an association between increased insulin resistance and the *PNPLA3* 148M allele was observed. This association was specifically present in HCV genotype 2 and it was not observed among HCV genotype 3 infected patients. Steatosis is tightly associated with insulin resistance [31,32]; however, the causal nature of this association remains to be fully elucidated. Insulin resistance has been commonly thought to be a risk factor for liver fat accumulation [33,34]. Nevertheless, new evidence suggests that hepatic steatosis might be pathogenically responsible for the development of insulin resistance [16,32,35]. The association of *PNPLA3* 148M allele with HOMA-IR in HCV infected subjects supports the hypothesis that insulin resistance may be interpreted as a consequence rather than a cause of hepatic steatosis. To date the role of the *PNPLA3* I148M variant on insulin resistance has been controversial, having some previous studies failed to find this association [8,13-15]; however our result is consistent with a recent study performed in normoglycaemic subjects from Taiwan [16] that reports 148M allele carriers having higher HOMA-IR levels. Further studies in larger cohorts are warranted to confirm this result.

Interestingly, we found that lower baseline viral load was associated with the *PNPLA3* 148M allele in HCV genotype 2 but not in HCV genotype 3 infected patients. This finding is novel [20], although difficult to interpret. HCV has been shown to exploit lipoprotein assembly and export pathways for the release of virions from infected hepatocytes [36-38]. Since *PNPLA3* 148M allele has been reported to influence lipid accumulation in the liver [39]*,* it is possible to hypothesize that *PNPLA3* 148M allele may impair both viral and lipoprotein release from infected hepatocytes resulting in lower baseline plasma viral load in HCV genotype 2 infection. In the presence of HCV genotype 3 infection, however, the intrahepatic impact of *PNPLA3* 148M allele on lipid particle production appears to be overridden by viral factors.

An important limitation of the present study is the relative small sample size of the HCV genotype 2 infected patients, as well as relative lower rate of *PNPLA3* 148M allele carriage in Northern Europeans as compared to previous reports from Southern Europe. In fact, even though steatosis has been widely reported to be associated with *PNPLA3* 148M allele [7,18-20], only a non-significant trend towards higher prevalence of steatosis was observed among *PNPLA3* 148M allele carriers in both HCV genotypes in the present study. However, in the analysis adjusted for confounders, the increased risk of steatosis reached marginal significance in genotype 2 but not 3 infected individuals, despite the threefold larger sample size of the latter group. This is in line with previous data showing an association between the *PNPLA3* I148M genotype and steatosis in the HCV genotype 2 but not in the HCV genotype 3 infected subjects [18,19]. Similarly, the relative small sample size of HCV genotype 2 infected patients in the present study may have contributed to the lack of association of the *PNPLA3* 148M allele with therapeutic outcome despite its effect on baseline viral load and insulin resistance. It is important to bear in mind that given the small sample size our results should be carefully interpreted due to the possibility of both false positive and negative associations. Therefore, this should be considered as a preliminary report and the results need to be confirmed in larger patient cohorts.

## Conclusions

Our results suggest an association between the *PNPLA3* 148M allele and insulin resistance as well as viral load in HCV genotype 2, but not genotype 3 infected individuals. Additional further genetic studies are required to elucidate the relationship between HCV infection, metabolic traits and *PNPLA3* I148M genetic variant.

## Competing interests

None of the authors have an association that might pose a conflict of interest.

## Authors’ contributions

KR, CM: equal contributions; responsible for data compilation, statistical modeling and writing of the manuscript. JW, SR: equal contributions; planning and performing of the study, data compilation, statistical modeling, corresponding authors, writing of the manuscript. MLa, CP, MAB, KH : planning and performing of the study, data compilation, statistical modeling, writing of the manuscript. MLi: responsible for genetic analyses. PBC, MF, NL, MRB, CP, KM, GN: clinical management of patients, planning and performing the treatment trial. All authors read and approved the final manuscript.

## Pre-publication history

The pre-publication history for this paper can be accessed here:

http://www.biomedcentral.com/1471-2350/13/82/prepub

## Supplementary Material

Additional file 1 ** Table S1.** Genotype and allele frequencies of the PNPLA3 I148M sequence variant in HCV genotype 2 and 3 individuals. Click here for file
