# Supplementary material for: PNPLA 3 I148M genetic variant associates with insulin resistance and baseline viral load in HCV genotype 2 but not in genotype 3 infection
Source: BMC Med Genet. 2012 Sep 14;13:82. doi: 10.1186/1471-2350-13-82 (PMC3495049; doi:10.1186/1471-2350-13-82)
Supplement: Additional file 1 — Table S1. Genotype and allele frequencies of the PNPLA3 I148M sequence variant in HCV genotype 2 and 3 individuals. [file 1471-2350-13-82-S1.docx]

**Supplementary table 1. Genotype and allele frequencies of the *PNPLA3* I148M sequence variant in HCV genotype 2 and 3 individuals.**

|  | **HCV GENOTYPE 2** | **HCV GENOTYPE 3** | **P value**† |
| --- | --- | --- | --- |
| **II (%)** | 56 (54)* | 159 (62)* |  |
| **IM (%)** | 43 (42) | 91 (36) |  |
| **MM (%)** | 4 (4) | 6 (2) |  |
| **Total** | 103 (100) | 256 (100) | 0.347 |
|  |  |  |  |
| **I (%)** | 155 (75) | 409 (80) |  |
| **M (%)** | 51 (25) | 103 (20) |  |
| **Total** | 206 (100) | 512 (100) | 0.205 |

Abbreviations: PNPLA3, patatin-like phospholipase domain-containing 3; HCV, hepatits C virus; II, individuals with two 148I alleles; MM, individuals with two 148M alleles; IM, heterozygotes.

*Frequencies are in Hardy-Weinberg equilibrium.

†P values were calculated using χ^2^ test
